# Advanced Nanosystems and Emerging Therapies: Innovations in Tuberculosis Treatment and Drug Resistance

**DOI:** 10.3390/pharmaceutics17111459

**Published:** 2025-11-12

**Authors:** Akhil Sharma, Vikas Sharma, Shivika Sharma, Sonu Sharma, Monu Sharma, Iyyakkannu Sivanesan

**Affiliations:** 1 School of Bioengineering and Biosciences, Lovely Professional University, Jalandhar 144411, Punjab, India; sharmaakhil177@gmail.com (A.S.); biotech_vikas@rediffmail.com (V.S.); shivikasharma25@gmail.com (S.S.); 2 Baba Nahar Biotech and Research, Bilaspur 174001, Himachal Pradesh, India; sharmasonu70462@gmail.com (S.S.); monusharma311998@gmail.com (M.S.); 3 Department of Environmental Health Science, Human and Eco Care Center, Konkuk University, 1 Hwayang-dong, Gwangjin-gu, Seoul 05029, Republic of Korea

**Keywords:** tuberculosis, nanomedicine, drug resistance, targeted drug delivery, host-directed therapy, CRISPR-Cas, nanoparticle vaccines

## Abstract

Tuberculosis (TB) remains a significant worldwide health challenge due to the limitations of conventional treatments and the rising incidence of drug-resistant *Mycobacterium tuberculosis* strains. This review consolidates the advancements in nanotechnology-based therapeutics, inhalable formulations, CRISPR–Cas tools, host-directed therapies (HDTs), and nanoparticle-based vaccine development aimed at enhancing TB management. Novel nanocarriers such as liposomes, solid-lipid nanoparticles (SLNs), dendrimers, and polymeric nanoparticles (NPs) offer enhanced bioavailability of drugs, sustained release, as well as targeted delivery to infected macrophages, thereby reducing systemic toxicity and dosing frequency. Inhalable nanomedicines provide localized delivery to the pulmonary site, enhancing the concentration of the drug at the primary site of infection. CRISPR–Cas technology is emerging as a transformative approach to disabling drug-resistant genes and enhancing diagnostic precision. HDTs, including agents like vitamin D and metformin, show potential in modulating host immune responses and enhancing pathogen clearance. Nanoparticle-based vaccines, including mRNA and antigen-conjugated platforms, aim to overcome the limitations of the BCG vaccine by enhancing antigen presentation and eliciting stronger, longer-lasting immunity. Collectively, these modalities mark a shift toward more personalized, effective, and less toxic TB therapies. However, challenges such as regulatory approval, safety, scalability, and accessibility remain. This review highlights the integrated potential of nanomedicine, gene editing, and immunomodulation to transform TB care and combat drug resistance, paving the way for more robust and durable treatment strategies.

## 1. Introduction

Tuberculosis (TB) emerged as the deadliest communicable disease globally, outpacing even HIV/AIDS in annual fatalities [1]. The causative agent, *Mycobacterium tuberculosis*, typically targets the lungs but can spread to virtually any organ, making it a complex and multifaceted illness. Despite effective treatment protocols available for decades, over a million people still die each year, primarily in low and middle-income countries, largely due to the appearance of drug-resistant strains, such as multidrug-resistant (MDR), extensively drug-resistant (XDR), and drug-resistant (TDR) [2].

The conventional six-month regimen for drug-susceptible tuberculosis combines drugs such as isoniazid, ethambutol, rifampicin, and pyrazinamide. However, its success is jeopardized by poor adherence, driven by lengthy treatment duration, significant side effects, and the high pill burden [3]. This often leads to incomplete treatment and subsequent resistance development. MDR-TB, considered resistant to both isoniazid and rifampicin, requires longer, more toxic second-line drugs for up to two years. XDR-TB and TDR-TB further exacerbate the situation by resisting additional drug classes, severely limiting treatment options, and burdening healthcare systems.

In response to these challenges, nanotechnology presents a transformative frontier in TB therapy [4]. A variety of nanosystems, such as liposomes, polymeric as well as solid lipid nanoparticles, dendrimers, nanogels, and metal-based complexes, are under active investigation for delivering anti-TB drugs [5]. These carriers can improve water solubility, sustain drug release, boost bioavailability, and importantly, enable targeted delivery to infected macrophages, the cellular niche where *M. tuberculosis* persists. By directing higher concentrations of drugs into infected cells, nanocarriers aim to reduce systemic side effects, lower dosing frequency, and potentially curb the emergence of further resistance [6].

Liposomes, spherical vesicles made of lipid bilayers, are highly promising carriers for delivering both water-soluble and fat-soluble anti-TB drugs [7]. Their similarity to cell membranes allows them to merge with host cells, promoting effective drug delivery. Liposomal formulations of rifampicin and isoniazid have been reported to achieve higher intracellular concentrations and stronger bactericidal activity against *M. tuberculosis*. In a similar vein, solid lipid nanoparticles (SLNs) and nanostructured lipid carriers (NLCs) have been developed with improved stability and drug-loading capabilities. These lipid-based systems enable controlled release, extended circulation periods, and enhance patient adherence [8].

Engineered polymeric nanoparticles made from polymers such as PLGA, chitosan, and alginate offer further advantages. They can be surface-modified with targeting ligands like mannose or folate to home in on macrophages, boosting drug specificity and uptake. Co-encapsulation of multiple drugs in one nanoparticle supports combined treatment regimens, lowering dose frequency and reducing the risk of resistance [9]. Dendrimers, highly branched, nanoscale polymers, allow multifunctionality through conjugation of drugs, targeting molecules, and imaging agents [10]. They have shown potential for delivering both conventional drugs and nucleic acids in TB therapy. Nanogels, which are hydrophilic polymer networks capable of swelling in response to stimuli, provide controlled and localized drug release, ideal for site-specific, timed therapy [11]. Beyond these drug delivery systems, a complementary treatment strategy involves Host-Directed Therapies (HDTs) [12]. By modulating the host’s immune system via pathways like autophagy, inflammation, or immune regulation, HDTs aim to improve pathogen clearance while limiting damage to tissue as well as resistance. Immunomodulatory agents such as vitamin D, statins, and metformin have shown potential in bolstering host immunity and improving treatment outcomes when paired with anti-TB drugs [13].

**Figure 1:**
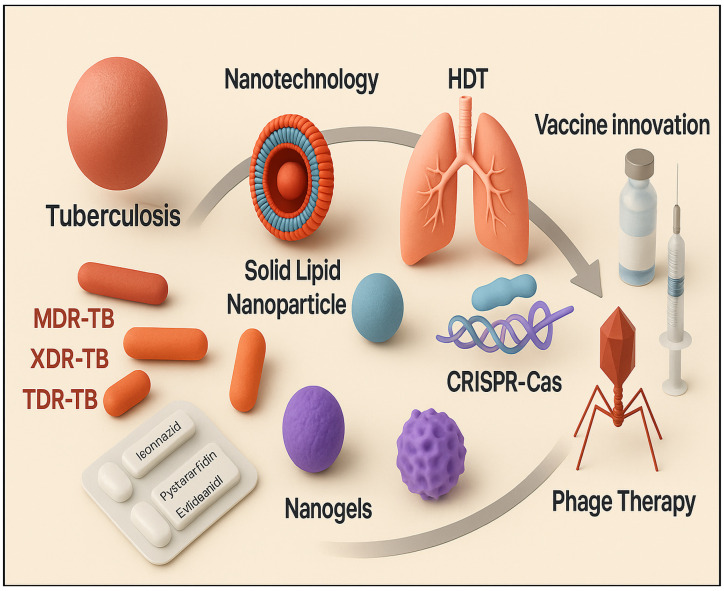
Integrated approaches in tuberculosis treatment combining conventional drugs with nanotechnology, gene editing, host-directed therapy, and vaccine innovation.

Emerging CRISPR–Cas technologies are offering revolutionary ways to combat *M. tuberculosis* by precisely targeting essential genes. In particular, CRISPR interference (CRISPRi) enables the specific silencing of genes involved in virulence and drug resistance, potentially rendering TB strains more susceptible to existing drugs [14]. Similarly, phage therapy, using bacteriophages to infect and destroy *M. tuberculosis*, is experiencing renewed focus (Figure 1). Specialized mycobacteriophages have demonstrated efficacy against multidrug-resistant TB, making them promising adjuncts or alternatives to antibiotics [15].

Vaccine innovation is another key front in TB control. While BCG remains the only licensed vaccine, its effectiveness varies widely [16]. A new generation of vaccines is underway, ranging from subunit and viral-vectored types to mRNA platforms. Nanoparticles are being harnessed not only as delivery vehicles and adjuvants to augment immune responses, but also in theranostic formats that combine therapy with monitoring, enabling real-time tracking of drug delivery and the progression of disease for tailored treatments [17]. These “theranostic” nanoparticles hold promise for personalizing TB therapy, improving efficacy while minimizing unnecessary exposure. Transitioning these nanotechnologies and advanced therapies from the lab to clinical practice poses challenges: scalable manufacturing, regulatory approval, safety profiling, and cost-effectiveness. This is particularly relevant in countries that have low- and middle-income where the burden of tuberculosis is highest [18]. Altogether, the combination of nanosystems, genome-editing technologies, phage-based treatments, and vaccine advancements marks a pivotal transformation in TB care. However, a concerted, multidisciplinary push from bench to bedside is essential to bridge innovation and implementation and move toward global TB elimination [19]. Despite numerous reviews addressing tuberculosis (TB) therapy and nanomedicine, most existing studies have focused either on classical anti-TB drug regimens or individual nanocarrier systems without integrating modern technological advances into a unified therapeutic framework. The present review distinguishes itself by providing a comprehensive, system-level overview of both established pharmacological principles and emerging technological innovations in TB management. Specifically, we integrate the recent progress in advanced nanoformulations (e.g., liposomes, polymeric and lipid nanoparticles, solid lipid carriers), stimuli-responsive nanosystems, and host-directed nanotherapies with novel enabling tools such as microfluidics-based drug screening, artificial intelligence-assisted optimization, and multi-omics-guided target identification. Furthermore, this review emphasizes simulation-based optimization of nanosystems, pharmacokinetic modeling, and multi-scale strategies bridging bench-to-bedside translation. By critically comparing these advancements to prior literature, our review highlights the transformative potential of nanotechnology and integrated bioengineering approaches in overcoming the limitations of conventional TB chemotherapy, providing a forward-looking roadmap for future research and clinical translation.

## 2. Pathogenesis and Immunology of Tuberculosis

The first step of infection occurs when an infected individual, with active tuberculosis (TB), releases infectious droplets and a second person inhales them. Their long half-life allows these droplets to stay in the air for a long time, and a single droplet can cause the disease process [20]. Most of the droplets are captured and neutralized in the upper respiratory system, but some of them get into the lungs. In this case, alveolar macrophages endeavor to phagocytize the *Mycobacterium tuberculosis bacilli* (MTB) by a mechanism that involves several surface receptors, such as mannose receptors, Toll-like receptors 2 and 4 (TLR2, TLR4), complement receptors, scavenger receptors, CD14, and surfactant protein A receptors [21]. Nevertheless, MTB has strategies to avoid being destroyed by disrupting phagosome-lysosome fusion, and thus it can survive and replicate inside macrophages. The second stage involves multiplication of the bacteria within the host cell until it ruptures and dies. This cellular destruction is an indication of the activation of monocytes and other immune cells in the infected area. When they reach the site, the monocytes differentiate into macrophages, and they also fail to eradicate the intracellular bacteria, which are lysed by the increasing bacterial load [22]. The third stage occurs about two to three weeks after infection and involves activation of adaptive immunity. The presentation of mycobacterial antigens causes T lymphocytes to migrate to the lungs and start to secrete cytokines, especially interferon-gamma (IFN-γ), which increases the bactericidal activity of macrophages. These stimulated macrophages secrete proinflammatory factors like IL-12, TNF-alpha, and IL-8, which assist in the development of a more potent immune response. At this stage, the growth of bacteria is slowed, and the host starts to acquire cell-mediated immunity.

**Figure 2:**
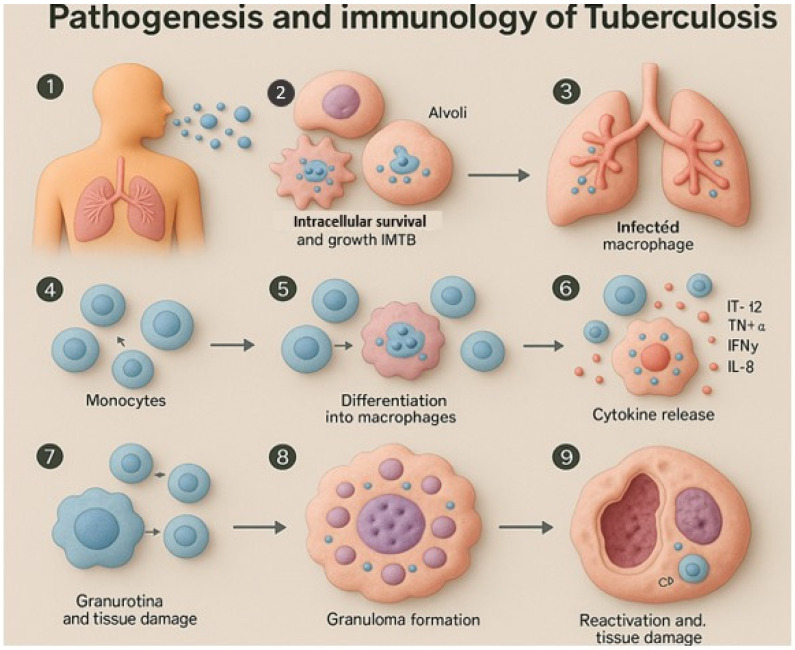
Stepwise illustration of tuberculosis pathogenesis and immune response, from droplet inhalation to granuloma formation and reactivation.

Over time, granulomas or tubercles form around the infected macrophages, enclosing the bacteria within a hypoxic and acidic environment that restricts further growth. This leads to a latent stage of TB, in which the host and pathogen reach a state of balance. Nevertheless, under conditions such as HIV infection, immunosuppression, malnutrition, or prolonged corticosteroid use, the granulomas can break down. When the core of a tubercle liquefies, it provides a nutrient-rich environment that facilitates rapid bacterial replication. This reactivation can result in extensive tissue necrosis surrounding the tubercular cavities [23]. Most TB infections are controlled by the immune system at this stage, but in some cases, the disease progresses further. CD4+ helper T cells and CD8+ cytotoxic T cells play central roles in the immune response to TB. CD4+ cells aid in enhancing macrophage activity through the secretion of IFN-γ and TNF, while CD8+ T cells can directly kill diseased macrophages and MTB by releasing cytotoxic molecules such as granulysin, perforin, and granzymes (Figure 2). Despite growing insight into the immune mechanisms involved in TB, the specific immune responses needed for a protective and effective vaccine remain incompletely understood [24].

## 3. Present Scenario for Tuberculosis Treatment

The common therapy regimen in both pulmonary and extrapulmonary tuberculosis is a long-term multidrug regimen of 6 to 12 months, depending on the severity and the kind of infection. Nevertheless, this long period presents major problems, such as the problem of patient non-compliance and failure to follow the prescribed regimen fully. These treatment failures may lead to the development of drug-resistant strains of *Mycobacterium tuberculosis* (MTB). The management of drug-resistant TB, such as multidrug-resistant (MDR-TB) and extensively drug-resistant TB (XDR-TB), is much harder and may need more complicated treatment regimens. The other significant barrier to TB management is the existence of non-replicating, dormant subpopulations of bacteria. The forms are caused by adverse intracellular conditions like nutrient deprivation, hypoxia, acidic pH, and accumulation of such compounds as nitric oxide (NO), carbon monoxide (CO), ascorbic acid, and granuloma formation [22]. These inactive bacilli have very specialized metabolic conditions and have a thick and lipid-laden cell wall, which makes them relatively impermeable to most of the conventional anti-TB medications. Although these latent bacilli are dormant over long periods, they may reactivate in the lifetime of an individual, leading to a recurrence of the active disease. Regrettably, no specific drug is available now that can target these non-replicating forms or kill them.

**Figure 3:**
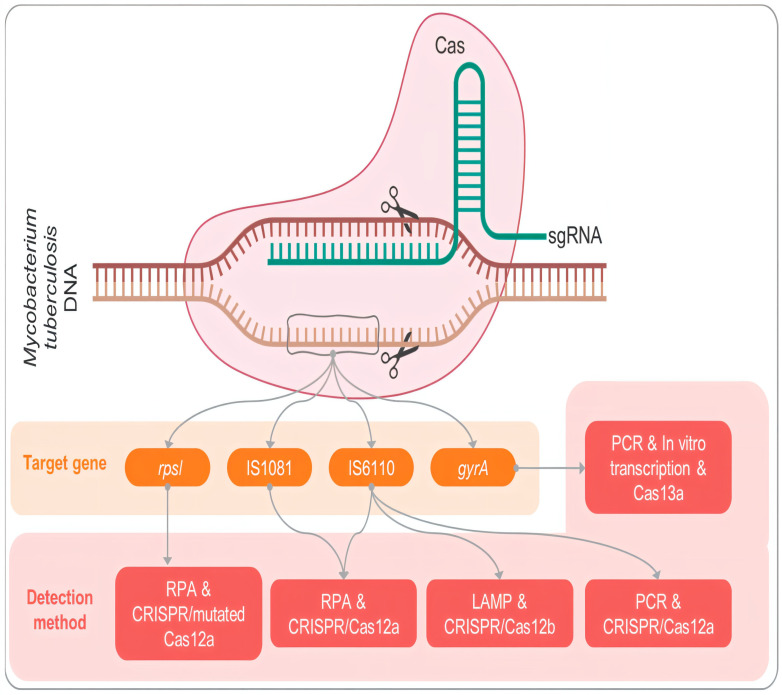
CRISPRCas-21 and gene editing in the management of tuberculosis. The schematic demonstrates the concept of CRISPR-Cas-based detection and targeting of *Mycobacterium tuberculosis* genes. The pink-colored patch represents the *M. tuberculosis* DNA, and the green items denote the single-guide RNA (sgRNA) and Cas enzyme complex, which are involved in gene identification and cleavage. The orange boxes represent specific target genes (e.g., rpsL, IS1081, IS6110, gyrA), and the red boxes denote the respective molecular detection schemes used for each of these target genes (e.g., RPA-CRISPR/Cas12a, LAMP-CRISPR/Cas12b, PCR-CRISPR/Cas13a). Arrows describe the direction of association between the gene and the methods. Gene targets (orange) and detection platforms (red), as well as the CRISPR components, are differentiated by color coding (green) [25].

To date, only ten anti-tuberculosis medications have received approval from the U.S. Food and Drug Administration. In India, the Directly Observed Treatment, Short-course (DOTS) strategy is employed to manage TB. This regimen includes a combination of four oral antibiotics, with the optional addition of injectable streptomycin, administered over six to twelve months [20]. In addition to the core first-line drugs, several other medications have shown potential against *M. tuberculosis* (Figure 3). For instance, clofazimine has demonstrated both anti-mycobacterial and immunosuppressive effects; amoxicillin interferes with bacterial cell wall synthesis; rifabutin inhibits RNA synthesis; and long-acting rifamycin, along with inhaled interferon-gamma and clarithromycin, which inhibits protein synthesis, are also employed in certain treatment protocols [23].

## 4. Emerging Technologies in Tuberculosis Treatment: A Problem–Solution Approach

Traditional TB therapy, characterized by long treatment time, undesirable side effects, and the growing number of drug-resistant *Mycobacterium tuberculosis*, is becoming ineffective [26]. Consequently, researchers are turning to cutting-edge nanosystems and novel therapeutic strategies to overcome these challenges. Below is an overview of the most promising approaches currently under investigation or development.

### 4.1. Conventional Antitubercular Therapy (ATT)

The traditional antitubercular therapy (ATT) has remained the primary treatment of the drug-sensitive *Mycobacterium tuberculosis* infections according to the WHO-recommended guidelines [27]. ATT is usually given in a 6-month treatment that consists of two phases: the intensive phase of 2 months, which involves the use of four first-line drugs (isoniazid, rifampicin, pyrazinamide, and ethambutol) to reduce bacterial load rapidly, and the continuation phase of 4 months during which isoniazid and rifampicin are used to kill any remaining bacilli [28]. All of the drugs have a certain mechanism: isoniazid interferes with the production of mycolic acids; rifampicin blocks the work of RNA polymerase; pyrazinamide is most effective in the acidic environment of phagosomes; ethambutol prevents the formation of the cell wall. Cure rates are more than 85% when the regimen is used appropriately. The conventional six-month regimen for drug-susceptible tuberculosis (DS-TB) comprises a two-month intensive phase with isoniazid, rifampicin, pyrazinamide, and ethambutol, followed by a four-month continuation phase with isoniazid and rifampicin, as recommended by the World Health Organization (WHO) and the Centers for Disease Control and Prevention (CDC). These first-line agents act synergistically by inhibiting mycolic acid synthesis (isoniazid, ethambutol), blocking RNA polymerase (rifampicin), and disrupting energy metabolism (pyrazinamide), thereby achieving rapid bacterial clearance and preventing relapse.

Nonetheless, this method has its limitations: it has long-term treatment, hepatotoxicity, neuropathy, inadequate bioavailability, and drug–drug interactions (especially with antiretrovirals) [29]. The result of poor adherence has resulted in the development of MDR-TB, XDR-TB, and even TDR-TB, which is increasingly becoming a threat to public health. The DOTS strategy was implemented to enhance adherence, but this has not completely solved these problems. Where there is drug resistance, second-line agents like fluoroquinolone and injectable aminoglycosides can prolong treatment to two years, however, at the cost of increased toxicity. Also, traditional ATT is not as effective in children, extrapulmonary, and TB-HIV co-infected patients [30]. All these shortcomings highlight the necessity of developing new TB drugs that are shorter, safer, more effective, and can overcome drug resistance, which has led to the recent increase in the number of studies on advanced nanosystems and new methods of treatment [31] (Table 1).

### 4.2. Nanocarrier-Based Drug Delivery Systems

Conventional anti-TB drugs suffer from poor aqueous solubility, limited intracellular accumulation, and systemic toxicity, leading to extended treatment duration and patient non-compliance. Addressing these pharmacokinetic limitations is essential for improving therapeutic success. Nanocarrier-based delivery systems, such as liposomes, solid lipid nanoparticles (SLNs), and polymeric nanoparticles, offer a promising solution to enhance drug solubility, prolong release, and enable macrophage-targeted delivery. Nanotechnology-powered drug delivery systems are revolutionizing tuberculosis treatment by overcoming many limitations of conventional antitubercular therapy [33]. These platforms use nanoscale carriers, typically between 1 and 1000 nm, to encapsulate anti-TB medications and deliver them directly to infected macrophages, where Mycobacterium tuberculosis resides [34]. Such drugs traditionally suffer from poor solubility, limited bioavailability, systemic toxicity, and inadequate penetration into granulomas or intracellular compartments. Nanocarriers offer targeted, controlled, and sustained drug release, enhancing effectiveness while reducing dose frequency and side effects.

Nanocarrier platforms are being studied in a broad spectrum [34]. Liposomes have phospholipid bilayers that may encapsulate hydrophilic and lipophilic drugs, shield them against degradation, and enhance the uptake by macrophages. Hydrophobic agents such as rifampicin have a better loading capacity, stability, and controlled release with solid lipid nanoparticles (SLNs) and nanostructured lipid carriers (NLCs) [35]. Polymeric nanoparticles based on biocompatible materials such as PLGA or chitosan enable programmable release profiles and customization (Table 2). Dendrimers, with their branched architecture and modifiable surfaces, allow for high drug payloads and precise targeting. Metallic nanoparticles, such as those containing silver or gold, demonstrate intrinsic antimicrobial properties, although concerns remain regarding their toxicity [36].

### 4.3. Exosomes as Emerging Nanocarriers in Tuberculosis Treatment

Exosomes have recently emerged as a promising frontier in nanomedicine for tuberculosis therapy. These nanosized extracellular vesicles (30–150 nm) secreted by various cell types naturally mediate intercellular communication by transferring proteins, lipids, and nucleic acids. Their intrinsic biocompatibility, stability, and ability to traverse biological barriers make them ideal candidates for drug and gene delivery. In the context of tuberculosis, exosomes derived from macrophages or dendritic cells can encapsulate anti-TB drugs, small interfering RNAs (siRNAs), or immunomodulatory molecules, enabling targeted delivery to *Mycobacterium tuberculosis*-infected cells while minimizing systemic toxicity. Recent evidence suggests that engineered exosomes can modulate immune responses by regulating macrophage polarization, enhancing antigen presentation, and restoring autophagic clearance pathways impaired during infection. The study comprehensively highlighted the therapeutic and diagnostic potential of exosome-based platforms in TB management. Despite their promise, large-scale isolation, standardization, and reproducibility remain major translational challenges. Overall, exosome-based nanocarriers represent a biologically inspired and clinically relevant direction for next-generation TB nanotherapies.

### 4.4. Inhalable Nanomedicine

Systemic administration of anti-TB drugs often fails to achieve sufficient concentrations at the primary site of infection—the lungs—while causing adverse effects elsewhere. To overcome this limitation, inhalable nanomedicines provide localized drug delivery directly to pulmonary tissues, enhancing therapeutic concentration at the infection site while minimizing systemic exposure. The use of nanomedicine as an inhalable drug is a modern and specific method of TB treatment because antitubercular drugs can be delivered to the lungs, which is the main locus of *Mycobacterium tuberculosis* infection [37]. This avoids the systemic circulation and the gut, and thus limits the side effects, increases drug bioavailability, and penetration into infected lung tissues and granulomas. Drugs in the size range 1–500 nm can be delivered to deep lung areas using nanocarriers like liposomes, polymeric nanoparticles, solid lipid nanoparticles (SLNs) and nanoemulsions in formulations of dry powder inhalers (DPIs), metered-dose inhalers (MDIs) or nebulized aerosols, and are taken up by alveolar macrophages in the lung to be released intracellularly over an extended period [38].

Inhalable formulations of key anti-TB agents, rifampicin, isoniazid, pyrazinamide, and newer drugs like bedaquiline, have demonstrated improved pulmonary availability, prolonged lung retention, and reduced systemic toxicity, which could lead to better efficacy and enhanced patient adherence. Moreover, functionalizing these nanocarriers with targeting ligands enhances macrophage uptake and helps overcome biological barriers. Preclinical studies in resistant TB models indicate a significant reduction in bacterial burden following inhalable delivery [39]. Several hurdles must be addressed: scalable production methods, formulation stability, device compatibility, and regulatory approval remain key challenges. Despite these obstacles, inhalable nanomedicine holds great promise as a non-invasive, patient-centric strategy that could reshape TB therapy by delivering targeted, efficient, and safer drug delivery straight to the lungs [40].

### 4.5. Gene Editing and CRISPR-Cas Technology

One of the greatest challenges in TB management is the emergence of multidrug-resistant (MDR) and extensively drug-resistant (XDR) strains due to spontaneous mutations in target genes. Novel gene editing tools, particularly CRISPR–Cas systems, have emerged as powerful instruments for combating drug resistance by selectively silencing or modifying virulence and resistance-associated genes in Mycobacterium tuberculosis. CRISPR–Cas systems are transforming tuberculosis research and treatment by offering precise genome manipulation of both *Mycobacterium tuberculosis* and host cells [41]. These molecular “scissors” enable targeted editing or silencing of genes, making it possible to disable bacterial genes essential for virulence, survival, or drug resistance, and to modulate host immune pathways such as macrophage activation and autophagy to bolster resistance to infection. In addition, CRISPR-based diagnostics, including platforms like SHERLOCK, DETECTR, and Cas13a systems, provide fast, sensitive, and specific detection of *M.tb* DNA from clinical samples, even in drug-resistant cases, facilitating early diagnosis and more personalized treatment [25].

While the technology remains largely experimental, its potential spans the development of targeted antimicrobials, genetically attenuated vaccines, and immune-directed therapies. Hurdles remain, including off-target effects, ethical considerations, delivery challenges, and regulatory barriers, before clinical translation can be realized. Overall, CRISPR–Cas technology offers a forward-looking and versatile toolkit for TB management, spanning diagnostics, therapeutics, and preventative measures, that could fundamentally reshape global TB control strategies. Recent advances have demonstrated tangible progress in CRISPR-based diagnostics and therapeutic proof-of-concept studies for tuberculosis. CRISPR–Cas12a and Cas13a platforms have been successfully employed for nucleic acid detection of Mycobacterium tuberculosis with high sensitivity and specificity, notably in assays such as SHERLOCK (Specific High-sensitivity Enzymatic Reporter unLOCKing) and DETECTR (DNA Endonuclease Targeted CRISPR Trans Reporter) [25]. These systems can identify Mtb DNA or drug-resistance-associated mutations within one hour and are being optimized for portable, point-of-care diagnostic use. On the therapeutic front, in vitro studies have utilized CRISPR interference (CRISPRi) to silence essential genes such as katG, inhA, and rpoB, validating the potential of CRISPR systems for targeted gene suppression and resistance modulation. Although no in vivo therapeutic trials have yet been completed, several animal-model-based feasibility studies are underway to explore the delivery of CRISPR components to infected macrophages and granulomatous tissues. Together, these emerging studies demonstrate that CRISPR–Cas tools are transitioning from conceptual innovation to applied translational research in TB diagnostics and therapeutics. Despite its transformative potential, one of the primary challenges in translating CRISPR–Cas technology into tuberculosis therapy lies in the efficient delivery of CRISPR components to intracellular *Mycobacterium tuberculosis* residing within granulomas. These lesions possess dense cellular and fibrotic barriers that restrict molecular transport, necessitating innovative delivery strategies. Both viral and non-viral systems are being explored to address this issue. Viral vectors such as lentiviruses and adeno-associated viruses (AAVs) offer high transfection efficiency but are limited by immunogenicity and packaging constraints. In contrast, non-viral carriers—including lipid nanoparticles, polymeric nanocarriers (PLGA, chitosan), dendrimer complexes, and bacteriophage-based delivery systems—provide safer and tunable alternatives capable of protecting guide RNAs and Cas proteins from degradation while enabling macrophage-targeted delivery. Recent research highlights that surface-engineered nanocarriers functionalized with mannose or peptides can penetrate granulomatous tissue and deliver CRISPR payloads directly to infected macrophages, improving gene-editing efficiency at the infection site. Incorporating such delivery strategies will be critical to realizing the therapeutic potential of CRISPR–Cas tools in TB management (Figure 3) [41].

### 4.6. TB Vaccines Based on Nanoparticles

The Bacillus Calmette–Guérin (BCG) vaccine provides variable protection, particularly in adults, and is insufficient to prevent pulmonary TB. To address these shortcomings, nanotechnology-assisted vaccines—using nanoparticles, virus-like particles, and mRNA platforms—are being designed to enhance antigen presentation, stimulate robust cellular immunity, and provide long-term protection. Tuberculosis (TB) vaccines based on nanoparticles are a new development in the search for more potent and long-lasting immunization methods, especially against the drawbacks of the conventional Bacillus Calmette-Guerin (BCG) vaccine. Although BCG provides partial protection against severe childhood TB, it is not very effective in preventing pulmonary TB in adults and is, in many cases, suboptimal [16].

Nanoparticle-based vaccine platforms aim to address these shortcomings by enhancing antigen delivery, presentation, and immune stimulation. These vaccines utilize engineered nanoparticles, such as liposomes, polymeric nanoparticles, virus-like particles (VLPs), and solid lipid nanoparticles, which encapsulate TB-specific antigens or DNA/RNA sequences encoding these antigens. The nanoscale size facilitates efficient uptake by antigen-presenting cells (APCs), such as dendritic cells and macrophages, ensuring targeted delivery to the immune system. The immunological rationale behind Host-Directed Therapies (HDTs) lies in their ability to modulate key host pathways that influence Mycobacterium tuberculosis survival and inflammation. Metformin, a well-known antidiabetic drug, activates the AMP-activated protein kinase (AMPK) pathway, which in turn enhances autophagy and promotes the phagolysosomal fusion necessary for intracellular bacterial clearance. Metformin also shifts macrophage polarization toward the M1 phenotype, enhancing proinflammatory cytokine production (e.g., TNF-α, IL-1β) and bactericidal activity. Similarly, statins exert immunomodulatory effects by inhibiting the mevalonate pathway, leading to reduced cholesterol accumulation in macrophage membranes, improved autophagic flux, and restoration of phagosomal maturation. Statins have also been shown to promote M1 polarization while limiting excessive inflammation by balancing M2 responses. Together, these mechanisms explain how repurposed agents like metformin and statins can potentiate host immune responses, reduce bacterial load, and improve therapeutic outcomes when combined with conventional anti-TB regimens ([42] (Table 3)).

Additionally, nanoparticles defend the antigens from degradation and permit for precise or sustained release, leading to prolonged immune activation. Some nanoparticles are also functionalized with adjuvants or surface ligands to further enhance the immune response by promoting both humoral (antibody-mediated) as well as cellular (T-cell-mediated) immunity, which is crucial for combating intracellular pathogens like *Mycobacterium tuberculosis* [43]. For instance, nanoparticles delivering fusion proteins like Ag85B, ESAT-6, and CFP10 have shown significant promise in inducing robust Th1-type immune responses in preclinical models [44].

**Figure 4:**
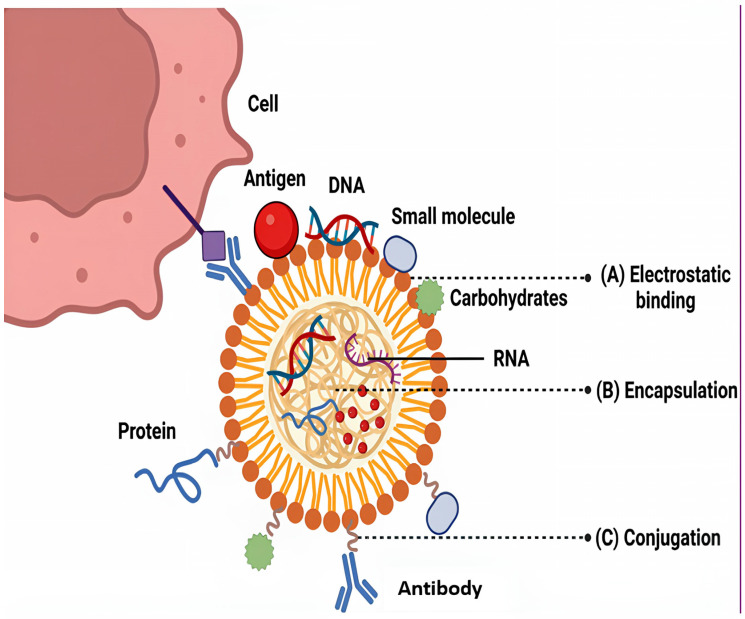
Nanoparticle-based vaccine for the treatment of tuberculosis [43].

Moreover, mRNA-based TB vaccines delivered via lipid nanoparticles, similar to those used in COVID-19 vaccines, are currently under investigation for their potential to rapidly stimulate protective immunity [45]. Despite encouraging preclinical and early clinical results, challenges such as large-scale production, stability, cost, and long-term safety assessments remain. Nevertheless, nanoparticle-based TB vaccines hold considerable potential to provide stronger, longer-lasting, and more targeted protection compared to traditional approaches, and could play a key role in future global TB eradication strategies. A comparative assessment of major nano vaccine platforms reveals distinct advantages and limitations in the context of tuberculosis immunization. Viral vector-based nano vaccines, such as adenoviral or modified vaccinia Ankara (MVA) systems, elicit strong cellular and humoral responses due to efficient antigen delivery and in vivo expression; however, their reuse can be limited by pre-existing vector immunity and higher production costs. In contrast, mRNA–lipid nanoparticle vaccines offer rapid design flexibility, high potency, and induction of both Th1 and cytotoxic T-cell responses, yet they face challenges related to cold-chain stability and cost-intensive formulation. Protein subunit nano vaccines—where TB antigens such as Ag85B, ESAT-6, or CFP10 are adsorbed or encapsulated in polymeric or lipid nanoparticles—are among the safest and most stable platforms with scalable manufacturing potential, though they often require potent adjuvants or booster doses to achieve durable immunity. Collectively, integrating these platforms with smart adjuvants and delivery nanocarriers may offer balanced immunogenicity, safety, and practical applicability for next-generation TB vaccines [46] (Figure 4).

### 4.7. Theranostic Nanoplatforms for Tuberculosis Detection and Therapy

Theranostics—systems combining therapy and diagnostics—represent a rapidly evolving frontier in tuberculosis management. These platforms enable real-time imaging, drug tracking, and localized therapy, improving treatment monitoring and personalization [47]. Metal–organic frameworks (MOFs), iron oxide nanoparticles, and gold–silica composites are under study for dual roles: serving as contrast agents (MRI, CT, fluorescence) while delivering anti-TB drugs. Theranostic nanoparticles enabling simultaneous rifampicin delivery and magnetic resonance imaging, allowing visualization of infection sites and drug distribution. Similarly, quantum-dot–based nanosystems functionalized with anti-TB drugs provide fluorescence-guided targeting of infected macrophages [48]. These advances open the path toward personalized and image-guided TB therapy, where clinicians can monitor therapeutic efficacy in real time.

### 4.8. Integrated Principles Underpinning Tuberculosis Therapy

In addition to these emerging nanotechnological and molecular approaches, the fundamental pharmacological principles that underpin tuberculosis therapy remain essential for optimizing therapeutic efficacy. These principles, summarized below, provide the mechanistic foundation for current and next-generation treatment strategies. The management of *Mycobacterium tuberculosis* is based on successful clearance of the pathogen in the host with minimization of the development of drug resistance and adverse drug reactions. This is typically achieved through a standardized multidrug regimen that accounts for the heterogeneity of bacterial populations within the host. The therapeutic approach comprises two distinct phases: an intensive phase aimed at rapidly decreasing the bacterial burden using a combination of isoniazid, rifampicin, pyrazinamide, and ethambutol, trailed by a persistence phase with isoniazid and rifampicin to eradicate persistent bacilli and prevent relapse [49,50].

The necessity of this extended six-month regimen arises from the pathogen’s intracellular lifestyle, slow replication, and capacity to enter dormancy within granulomatous structures, which present challenges for drug penetration. The possibility of interruption in treatment, causing multidrug-resistant (MDR) or extensively drug-resistant (XDR) strains, highlights the importance of strategies like directly observed treatment short-course (DOTS) to guarantee compliance [51,52]. Additionally, emerging paradigms emphasize improved drug delivery to infected niches and host-targeted interventions to enhance therapeutic efficacy.

Combination chemotherapy remains the cornerstone of TB management. Multidrug chemotherapy is still one of the foundations of TB treatment. A combination of agents with different mechanisms of action is necessary to inhibit the development of resistant subpopulations. Monotherapy is linked with the fast development of resistant mutants, leading to treatment failure and emergence of MDR or XDR strains [53,54]. A combination of four drugs (isoniazid, rifampicin, pyrazinamide, and ethambutol) minimizes the risk of developing resistance, guarantees the strong bactericidal effect, and facilitates positive clinical outcomes [55]. The method also minimizes the chances of relapse, which further justifies its use in the existing TB control procedures worldwide.

Targeting heterogeneous bacterial populations within the host is another guiding concept. Effective TB therapy must account for the physiological diversity of *M. tuberculosis* populations within the host. The pathogen exists in actively replicating, slowly replicating, and dormant states, primarily within alveolar macrophages and granulomas [56,57]. These structures, while confining the infection, also shield the bacteria from antibiotics and immune clearance. Active bacilli are more responsive to antimicrobials, whereas dormant forms demonstrate significant tolerance. Therefore, the intensive phase targets the metabolically active bacilli, while the continuation phase is intended to eradicate the persistent population, minimizing the risk of disease reactivation [58].

Extended treatment duration distinguishes TB therapy from most bacterial infections due to the organism’s slow replication and intracellular persistence. The prolonged duration of TB treatment distinguishes it from other bacterial infections. *M.tb* grows slowly and can persist latently, necessitating extended therapy to ensure bacterial clearance and to prevent relapse or resistance development [47,56]. Clinical evidence supports that shortening the treatment period without fully eradicating the infection compromises long-term outcomes. Consequently, patient education and adherence support mechanisms remain critical components of TB control strategies.

Optimization of drug delivery and bioavailability is central to improving treatment success. Conventional TB therapies are often limited by inadequate drug penetration into infected lung tissues and granulomas. The limitations have led to the emergence of new drug delivery methods that are geared towards increasing bioavailability and targeting of therapeutics. Systems based on nanocarriers, including liposomes, polymeric nanoparticles, and solid lipid nanoparticles, have demonstrated potential in enhancing the solubility, stability, and intracellular uptake of drugs [58,59]. These systems are used to release drugs over a long period of time and to deliver them to macrophages, thereby enhancing the treatment process.

Additionally, pulmonary delivery platforms (e.g., dry powder inhalers, nebulized formulations) offer localized deposition of anti-TB agents directly at the site of infection, increasing therapeutic efficacy and reducing systemic toxicity [60]. Such strategies are especially pertinent in cases of drug-resistant TB and represent a significant advancement in treatment delivery.

Adherence and patient compliance remain decisive for successful outcomes. Given the length and complexity of TB therapy, patient compliance is a major determinant of treatment success. Poor adherence increases the risk of incomplete bacterial eradication and promotes resistance [26]. The DOTS strategy, wherein healthcare providers supervise treatment administration, has been instrumental in improving completion rates and curbing resistance emergence [61]. Nonetheless, sustained investment in patient education, community engagement, and support infrastructure remains essential, particularly in high-burden, resource-limited settings.

Finally, host-directed therapeutic strategies represent an integrative approach that complements antimicrobial action. Host-directed therapies (HDTs) represent an emerging adjunct to standard antimycobacterial regimens. Rather than directly targeting the pathogen, HDTs aim to bolster host immunity, enhance pathogen clearance, and reduce immunopathology. These approaches include the modulation of autophagy, phagosome maturation, and cytokine responses to improve macrophage function and limit tissue damage [61,62].

Repurposed agents i.e., statins, vitamin D, metformin, and drugs that are anti-inflammatory, are under investigation for their immunomodulatory potential. Early clinical and preclinical studies suggest that HDTs may shorten treatment duration, reduce the emergence of resistance, and improve clinical outcomes when used alongside conventional therapy [63]. Together, these interconnected principles form the conceptual framework linking classical multidrug chemotherapy with cutting-edge nanotechnology and molecular innovations, underscoring the translational continuum from traditional regimens to personalized, next-generation tuberculosis therapy.

### 4.9. Microfluidics-Based Platforms for TB Drug Discovery and Nanomedicine Development

Recent advances in microfluidics have transformed the preclinical evaluation of tuberculosis (TB) therapeutics by enabling precise, high-throughput, and physiologically relevant models for drug screening, formulation optimization, and pathogen–host interaction studies [64]. Microfluidic systems, often referred to as “lab-on-a-chip” technologies, integrate microscale fluid channels, valves, and sensors to mimic the dynamic in vivo environment under controlled conditions while requiring minimal reagent volumes and time [65].

#### 4.9.1. Application in Anti-TB Drug Discovery

Microfluidic devices allow rapid screening of anti-tubercular compounds against both replicating and non-replicating *Mycobacterium tuberculosis* under controlled gradients of oxygen, nutrients, or drugs, thereby simulating the hypoxic and heterogeneous microenvironments found in granulomas [66]. For instance, developed a microfluidic “granuloma-on-chip” system that recreated human lung-like infection microenvironments to test drug efficacy against intracellular TB. These platforms facilitate real-time monitoring of bacterial growth, biofilm formation, and drug penetration using fluorescence and impedance-based sensors [67].

#### 4.9.2. Role in Nanomedicine Formulation and Testing

Microfluidics also provides unparalleled control over nanoparticle synthesis parameters such as flow rate, temperature, and mixing time, ensuring reproducible particle size distribution and drug loading efficiency. Anti-TB nanocarriers such as PLGA nanoparticles and liposomes have been fabricated via continuous-flow microreactors with improved monodispersity compared to bulk mixing methods [68]. Moreover, organ-on-chip systems integrating alveolar epithelial and macrophage cells enable simultaneous assessment of nanocarrier uptake, cytotoxicity, and intracellular drug delivery under shear stress conditions mimicking pulmonary physiology.

#### 4.9.3. Advantages

Microfluidics-based methods significantly reduce experimental time and resource consumption while enhancing precision and scalability. They offer a bridge between in vitro cell cultures and animal models, accelerating preclinical testing and guiding the design of inhalable and macrophage-targeted nanoformulations. The integration of microfluidics with 3D bioprinting and AI-assisted imaging holds future promise for automated TB drug discovery and personalized nanotherapeutic screening [69].

## 5. Mechanism of Action of Various Antibiotics and Medicines Used for Tuberculosis (TB) Treatment

*Mycobacterium tuberculosis* causes tuberculosis (TB), which remains one of the greatest killers of infectious diseases in the world. The unique characteristics of the pathogen, a lipid-rich cell wall, slow replication rate, and ability to enter a dormant state make it hard to treat successfully [64]. Conventional therapy is based on a regimen of first- and second-line anti-TB medication, which attack various facets of the mycobacterial cell or its metabolism [70]. Combining these drugs is important to kill the bacteria, avoid relapse, and minimize the chances of resistance. The World Health Organization’s six-month short-course treatment is based on four first-line antibiotics: isoniazid (INH), rifampicin (RIF), pyrazinamide (PZA), and ethambutol (EMB) [71].

The prodrug isoniazid is converted by the katG-encoded catalase-peroxidase to an active form that inactivates the enzyme InhA, which impairs the production of mycolic acids and weakens the cell wall. Rifampicin binds the β-subunit of DNA-dependent RNA polymerase (encoded by rpoB), preventing transcription and killing both replicating and dormant bacilli, though mutations in the rpoB gene are important determinants of resistance [72]. Pyrazinamide is converted by pyrazinamidase (pncA) into pyrazinoic acid, which accumulates under acidic conditions in phagosomes, disrupting membrane potential and energy metabolism [73]. Ethambutol, a bacteriostatic agent, inhibits the arabinosyl transferases encoded by the emb*CAB* operon, preventing arabinogalactan synthesis and weakening the cell wall [74]. Together, these drugs attack multiple critical pathways within *M. tuberculosis*, enabling an effective and synergistic treatment regimen that underpins the success of short-course therapy.

In patients with MDR-TB or extensively drug-resistant TB (XDR-TB), second-line and novel antitubercular agents are used. These are streptomycin, fluoroquinolones (levofloxacin, moxifloxacin), aminoglycosides (amikacin, kanamycin), bedaquiline, delamanid, linezolid, clofazimine, and pretomanid [75]. The earliest antibiotic to be used against TB is streptomycin, an aminoglycoside which interacts with the 30S ribosomal subunit of the bacteria [76]. This binding leads to inaccurate reading of mRNA during translation, leading to the synthesis of non-functional proteins. Streptomycin is bactericidal, especially against extracellular organisms, but its use has decreased because of the emergence of resistance and the introduction of less toxic alternatives.

Fluoroquinolones, e.g., levofloxacin and moxifloxacin, are used to inhibit bacterial DNA gyrase and topoisomerase IV, which are enzymes that maintain DNA supercoiling and replication. These enzymes are inhibited, leading to the damage of DNA and halting the division of the bacterial cells [77]. These are highly bactericidal drugs, and they are part of MDR-TB regimens, but they may develop resistance by mutating the gyrA and gyrB genes. Streptomycin, amikacin, and kanamycin are aminoglycosides that act by binding to the 30S ribosomal subunit and inhibiting protein synthesis. They are restricted by nephrotoxicity and ototoxicity, although they are applied in resistant TB cases where no superior options are available [78]. A diarylquinoline, bedaquiline, is a TB breakthrough in therapy. It acts on the c-subunit of ATP synthase, which is required in the production of ATP in *M. tuberculosis* [73].

Inhibition of ATP synthesis starves the bacteria of energy, leading to cell death. Bedaquiline is highly effective against MDR-TB and has a long half-life, allowing intermittent dosing. However, it must be used with caution due to the potential for QT interval prolongation and cardiotoxicity. Delamanid, a nitro-dihydro-imidazooxazole derivative, inhibits the synthesis of mycolic acid, affecting cell wall integrity. Additionally, it interferes with the electron transport chain, leading to a reduction in ATP synthesis [79]. Delamanid is useful against both replicating and non-replicating bacilli and is used in combination therapy for MDR- and XDR-TB. Linezolid, an oxazolidinone, prevents the synthesis of bacterial protein by binding to the 50S subunit of the ribosome, stopping the formation of the initiation complex. Though originally developed for Gram-positive infections, linezolid has shown significant activity against resistant TB strains [80]. It is used as a reserve drug due to its hematologic toxicity and the potential for neuropathy with long-term use.

Clofazimine, a riminophenazine dye, binds to the mycobacterial DNA and interferes with transcription and replication. It also induces the production of reactive oxygen species (ROS), which disrupts the cell membrane and causes bacterial death [81]. Clofazimine is often used in combination regimens for MDR-TB, especially when options are limited. Pretomanid, another nitroimidazole like delamanid, works by releasing nitric oxide under anaerobic conditions, damaging bacterial respiratory components. It also inhibits mycolic acid synthesis, affecting cell wall structure [82]. Pretomanid is part of the BPaL regimen (bedaquiline, pretomanid, and linezolid), a potent combination for the treatment of highly resistant TB forms.

**Figure 5:**
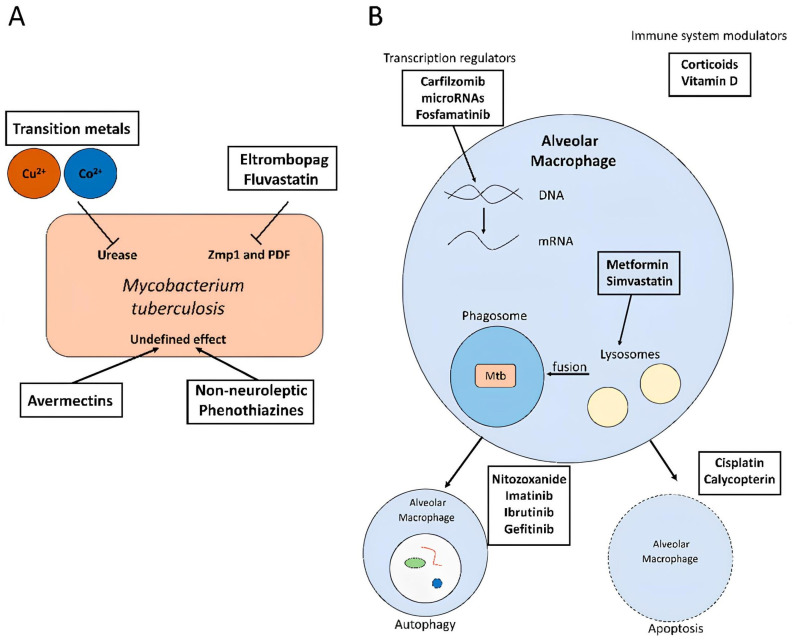
*M. tuberculosis* Mechanism of action of different antibiotics and adjunct compounds in the treatment of tuberculosis (TB). Without neuroleptics (**A**), Transition metals (Cu^2+^ and Co^2+^ ), and compounds like elthrombopag, fluvastatin, avermectins, and non-neuroleptic phenothiazines, *M. tuberculosis* acts directly by disrupting essential bacterial enzymes (urease, Zmp1, PDF), modulating bacterial metabolism and survival. Host-directed therapeutic approaches attack the alveolar macrophage (**B**). The following can modify gene expression, including transcription regulators (carfilzomib, microRNAs, fosfamatinib), phagosome-lysosome fusion with metformin and simvastatin, autophagy with nitazoxanide, imatinib, ibrutinib, and gefitinib, and apoptosis with cisplatin and calycopterin. Corticosteroids and vitamin D are immune modulators that help the host’s immune system against *M. tuberculosis*. [85].

The success of TB therapy relies not only on the potency of individual drugs but also on their synergistic use in combination regimens. By targeting various pathways, including DNA replication, RNA synthesis, protein synthesis, energy production, and cell wall biosynthesis, these medications collectively ensure the eradication of different bacterial populations, including actively replicating, dormant, and intracellular forms [83]. This multi-targeted approach is essential for preventing the development of resistance, achieving high cure rates, and reducing transmission. However, the emergence of drug-resistant strains necessitates continued research into newer drugs, combination strategies, and advanced drug delivery systems such as nanocarriers to improve therapeutic outcomes in TB management (Figure 5) [84].

## 6. Factors Affecting the Treatment of TB

Successful tuberculosis (TB) management relies on a complex interplay of biological, social, pharmacological, and clinical factors that shape drug effectiveness, treatment obedience, and the risk of drug resistance [86]. Biologically, co-morbidities such as HIV infection or immunosuppression influence both immune response and drug pharmacokinetics. Social determinants, including stigma, poverty, limited education, and lack of social support, can hinder patients’ ability to adhere to the lengthy treatment regimen [87]. Pharmacologically, variations in drug levels, side effects, and interactions, particularly in patients on antiretrovirals or those with co-existing conditions, can compromise therapy. Clinically, factors like age, disease severity, and sputum conversion at two months serve as early predictors of treatment success (Table 4). Recognizing and addressing these diverse influences is essential for optimizing individualized treatment, improving outcomes, and reducing the emergence of resistant TB strains [88].

### 6.1. Drug Resistance

Drug-resistant *Mycobacterium tuberculosis* strains are one of the largest obstacles to the successful treatment of TB. Resistance is either primary, when the patient has a resistant strain directly, or acquired, resulting from improper or incomplete treatment [94]. Multidrug-resistant TB (MDR-TB) is resistance to isoniazid and rifampicin, the most powerful first-line drugs. Extensively drug-resistant TB (XDR-TB) is resistance to fluoroquinolones and one or more second-line injectable drugs, seriously complicating the treatment [95]. MDR-TB and XDR-TB treatment is considerably more difficult: it involves longer regimens, more toxic and expensive drugs, and lower success rates compared to drug-sensitive TB. These resistant forms thrive when drug management is poor or when patients discontinue their treatment prematurely, underscoring the critical importance of adherence and robust healthcare systems [96]. Clinical studies from India and South Africa have shown that patients with previous incomplete treatment courses have over 10-fold higher risk of MDR-TB recurrence. For example, a 2023 WHO surveillance in Mumbai revealed 25% MDR-TB among previously treated patients.

To overcome this, rapid molecular diagnostics (e.g., GeneXpert MTB/RIF Ultra) and whole-genome sequencing help in early detection of resistant strains, allowing personalized regimens such as BPaL (bedaquiline-pretomanid-linezolid). Regular treatment monitoring and drug-susceptibility testing are vital to minimize amplification of resistance (Table 5).

### 6.2. Patient Compliance and Adherence

Adherence to TB therapy is crucial for the success of the treatment and to prevent the expansion of resistance. The standard TB regimen involves multiple antibiotics taken for at least six months, which can be burdensome for many patients. Side effects, long treatment duration, pill burden, lack of understanding of the disease, and socio-economic hardships all contribute to poor adherence [97]. Incomplete or irregular treatment allows bacteria to persist and mutate, leading to drug resistance and treatment failure. Directly Observed Treatment, Short-course (DOTS) is often employed to improve adherence [93]. In a clinical case study in Sub-Saharan Africa, adherence improved by 40% when community health workers directly supervised treatment and offered nutritional incentives.

Digital adherence technologies (99DOTS, smart pillboxes), patient education, and socio-economic support significantly reduce non-compliance. Counseling and community engagement are now considered integral to TB control (Table 5).

### 6.3. HIV Co-Infection

HIV-positive individuals are also at higher risk of developing active TB due to weakened immune systems [98]. Co-infection with TB alters the clinical presentation of TB, increases the risk of disseminated disease, and complicates treatment due to drug–drug interactions between the TB drugs and antiretrovirals, particularly rifampicin. HIV-TB co-infected patients require careful coordination of both treatment regimens, often with adjusted dosages and enhanced monitoring for adverse effects and overlapping toxicities [99]. Co-infected patients require staggered therapy initiation; ART is generally started 2–8 weeks after TB treatment initiation to prevent immune reconstitution inflammatory syndrome (IRIS). For example, in the SAPIT trial (2010, South Africa), early ART initiation reduced mortality by 56%. Strategy includes integrating TB–HIV clinics and using rifabutin instead of rifampicin minimizes drug–drug interactions (Table 5).

### 6.4. Pharmacokinetic and Pharmacodynamic Variability

Effective tuberculosis (TB) treatment depends on adequate drug levels inside the body, a process influenced by absorption, distribution, metabolism, and elimination [100]. Individual variations in genetics, age, body weight, nutritional status, and liver or kidney function can significantly affect blood concentrations of anti-TB medications. Insufficient drug levels may lead to suboptimal outcomes, toxicity, or the emergence of resistance. As a result, personalized dosing and therapeutic drug monitoring (TDM), which comprises the measurement of drug concentrations in the blood and adjusting dosages accordingly, may be necessary to ensure each patient achieves optimal therapeutic exposure [101] (Table 5).

### 6.5. Comorbid Conditions

Healthy treatment outcomes in tuberculosis (TB) are often compromised by coexisting conditions like diabetes, chronic liver or kidney disease, and malnutrition [102]. Diabetes weakens immune defenses and slows clearance of *M. tuberculosis*, frequently necessitating longer courses of therapy, while malnutrition diminishes immune response and interferes with drug metabolism, both linked to poorer treatment outcomes and higher relapse rates [103]. Chronic kidney and liver diseases also negatively affect prognosis, demanding dosage adjustments and close monitoring, with evidence showing these comorbidities predict lower success rates in multidrug-resistant TB cases [104]. Addressing these underlying health issues is vital to improving treatment efficacy and reducing TB relapse. Studies have shown that diabetic TB patients have 1.5–3× higher relapse rates. Integrated diabetes–TB screening programs (as piloted in Indonesia, 2022) improved glycemic control and treatment success (Table 5).

Intervention: Routine glucose monitoring and adjunct use of metformin can enhance macrophage function and improve TB outcomes.

### 6.6. Mycobacterial Load and Infection Site

The effectiveness of TB treatment is significantly influenced by the initial bacterial load and the location of infection. Pulmonary TB, particularly when there is a high burden of bacteria, often responds more slowly than extrapulmonary forms [105]. Infections in areas like the brain, spine, or bones are especially challenging, as drugs have difficulty penetrating these tissues. Moreover, *M. tuberculosis* that enters a dormant or slow-growing state, typically found in hypoxic or nutrient-poor environments within granulomas, is far less susceptible to standard antibiotics, contributing to persistent infections and relapse [106] (Table 5).

Clinical case: In CNS-TB, drug penetration across the blood–brain barrier is poor; thus, high-dose isoniazid or levofloxacin is employed.

Solution: Liposomal or intranasal nanocarrier formulations are being investigated to improve drug reach to extrapulmonary sites.

### 6.7. Socioeconomic and Environmental Factors

Living conditions such as poverty, overpopulation, and incomplete access to healthcare are significant barriers to effective tuberculosis (TB) control [107]. These factors can lead to deferred diagnosis, insufficient treatment, and decreased patient adherence. For instance, individuals in impoverished settings often face challenges like malnutrition and poor housing, which can weaken the immune system and hinder recovery. Overcrowded environments facilitate the rapid spread of TB, while a lack of healthcare access can prevent timely diagnosis and treatment [108]. Additionally, the stigma associated with TB may discourage individuals from seeking care, exacerbating the situation. Addressing these social determinants through improved living conditions, enhanced healthcare access, and public health initiatives is crucial for effective TB control and prevention [109] (Table 5).

### 6.8. Healthcare System Limitations

The effectiveness of tuberculosis (TB) treatment is heavily influenced by the efficiency and capacity of the healthcare system. Factors such as delayed diagnosis, medication stock-outs, inadequately trained healthcare personnel, and poor patient follow-up can significantly hinder treatment success [110]. For instance, delays in diagnosis can lead to prolonged infectiousness and increased transmission rates. Stock-outs of essential TB medications disrupt treatment regimens, potentially leading to drug resistance [111]. Untrained healthcare workers may mismanage cases, resulting in suboptimal treatment outcomes. Additionally, inadequate patient follow-up can cause interruptions in treatment, increasing the risk of relapse and resistance development [112]. Addressing these issues by strengthening diagnostic facilities, ensuring a consistent supply of quality medications, and providing comprehensive training for healthcare professionals is crucial to improving treatment success and minimizing resistance development. For example During COVID-19, delayed TB diagnosis led to 18% increase in untreated cases globally (WHO 2022) (Table 5).

Measures: Strengthening primary health infrastructure, digital reporting systems, and uninterrupted drug supply chains improve case detection and continuity.

### 6.9. Adverse Drug Reactions

Many anti-tuberculosis (TB) medications can cause side effects that may deter patients from adhering to their treatment regimens [113]. For instance, isoniazid and rifampicin are known to have hepatotoxic effects, potentially leading to liver damage. Ethambutol can cause optic neuritis, resulting in blurred vision or color blindness, while streptomycin may lead to ototoxicity, affecting hearing [114]. These adverse reactions can discourage patients from continuing therapy, especially if not properly managed. Therefore, educating patients about potential side effects, conducting routine monitoring of liver function and vision, and providing supportive care are essential strategies to mitigate these issues and enhance treatment adherence [115] (Table 5).

### 6.10. Host Immune Response

The immune system of the body plays an important role in monitoring and eliminating tuberculosis (TB) infections [116]. Individuals with compromised immune systems, such as those living with HIV and AIDS or undergoing immunosuppressant treatments, are at a higher risk for the progression and recurrence of TB [117]. In contrast, a strong immune response facilitates more rapid clearance of the bacteria. To enhance TB treatment outcomes, especially in immunocompromised patients, researchers are investigating host-directed therapies (HDTs). These therapies are intended to regulate the host immune response to enhance the host’s immune defenses against TB, as an adjunct to standard antimicrobial therapies. HDTs are studied as adjuncts to existing TB therapy to enhance clinical outcomes by stimulating the immune response, reducing inflammation, and avoiding reactivation of latent TB infection [118] (Table 5).

### 6.11. Delay in Treatment Initiation and Diagnosis

Early detection and prompt initiation of appropriate therapy are critical for effective tuberculosis (TB) treatment [119]. Adjournments in diagnosis and treatment can lead to disease progression, increased transmission, and poorer outcomes. Factors contributing to these delays include a lack of awareness, diagnostic challenges, and limited access to medical services. While rapid molecular diagnostic tools like GeneXpert have improved early detection, access remains limited in many regions due to infrastructure constraints, underfinancing, and logistical challenges [120]. Addressing these barriers is essential to ensure timely diagnosis and treatment initiation, ultimately improving TB control efforts (Table 5).

### 6.12. Drug Interactions and Polypharmacy

Many TB patients, especially those with comorbidities, are on multiple medications [121]. Drug interactions, especially involving rifampicin, can affect the efficacy of treatments for co-existing conditions such as HIV, diabetes, or epilepsy [122]. This necessitates careful drug selection, dose adjustment, and regular monitoring to avoid adverse events and therapeutic failure. Rifampicin reduces efficacy of oral contraceptives and warfarin by CYP3A4 induction (Table 5).

Management: Drug–drug interaction checklists and pharmacist-led medication reviews help prevent therapeutic failure in co-treated patients.

**Table 5:** Strategies to overcome major factors affecting TB treatment success.

S. No.	Factor	Key Strategies/Methods to Overcome	Reference
1	Drug resistance	Rapid molecular diagnostics (e.g., GeneXpert, WGS) individualized drug-susceptibility testing & tailored regimens Strengthening DOTS/monitoring to prevent resistance amplification	[123]
2	Patient compliance/adherence	Digital adherence technologies (smart pillboxes, text reminders) Community-based DOT, peer support, counseling Socio-economic support (food, transport) to reduce drop-out	[124]
3	HIV co-infection	Integrated TB–HIV clinics Early ART initiation according to guidelines Drug–drug interaction management (e.g., rifabutin vs. rifampicin)	[125]
4	PK/PD variability & host pharmacogenetics	Therapeutic drug monitoring (TDM) Genotype-guided dosing (e.g., NAT2 for INH) Adjusting drug doses in special populations (renal/hepatic impairment)	[126]
5	Comorbid conditions (diabetes, malnutrition)	Routine screening of comorbidities (DM, HIV, liver disease) Nutritional support & glycaemic control integration Host-directed adjunct therapies (e.g., metformin in diabetics)	[127]
6	Mycobacterial load & infection site (extrapulmonary, cavities)	Imaging/focal assessment (CT, MRI) for cavitary/extrapulmonary disease Extended/adjusted treatment duration/regimen in hard-to-reach sites Novel drug delivery (liposomal, intranasal) in future	[128]
7	Socio-economic & environmental factors	Social protection schemes (transport subsidy, food packages) Community awareness & stigma reduction programs decentralized care closer to patient homes	[129]
8	Healthcare system limitations	Strengthening primary health infrastructure Ensuring uninterrupted drug supply, quality diagnostics Digital health systems & case-tracking	[130]
9	Adverse drug reactions (ADRs)	Routine monitoring of liver/lab parameters Early recognition & management of ADRs (with hepatoprotective adjuncts) Tailoring therapy in high-risk patients	[131]
10	Host immune response	Screening for vitamin D deficiency, immunomodulators Host-directed therapies (vitamin D, statins, metformin) under investigation Addressing underlying immunosuppression (HIV, malnutrition)	[132]
11	Delay in diagnosis/treatment initiation	decentralized screening (mobile units, outreach) Rapid diagnostics and immediate linkage to care Public–private partnerships to reduce delay	[132]
12	Drug interactions & polypharmacy	Medication review & pharmacist involvement Standard checklists for drug–drug interactions (especially rifampicin, ART, warfarin, contraceptives) Adjust regimens accordingly	[133]

## 7. Conclusions and Future Scope

This study demonstrates that tuberculosis remains a critical health concern globally, with increasing complications due to multidrug-resistant (MDR), extensively drug-resistant (XDR), and drug-resistant (TDR) strains. Traditional treatment regimens, although foundational, are significantly limited by extended duration, side effects, poor adherence, and failure to eliminate dormant bacilli. The integration of nanotechnology in TB treatment introduces innovative drug delivery systems, including liposomes, polymeric nanoparticles, dendrimers, and solid lipid nanoparticles, which enhance intracellular drug delivery, improve pharmacokinetics, reduce toxicity, and support patient compliance. Inhalable nanomedicines, delivering drugs directly to the lungs, offer site-specific action and reduced systemic burden. The study also highlights the promising application of CRISPR–Cas systems, enabling precise targeting of *M. tuberculosis* genes associated with virulence and resistance. Host-directed therapies, including repurposed agents like vitamin D and metformin, modulate immune pathways such as autophagy and inflammation, assisting in bacterial clearance while reducing tissue damage. Although numerous nanocarrier systems have demonstrated promising preclinical efficacy in tuberculosis models, their clinical translation remains limited. A few platforms, such as liposomal amikacin (Arikayce^®^) and inhalable nanoparticle formulations of rifampicin and isoniazid, have progressed to early-phase clinical evaluations, primarily assessing safety and pulmonary deposition profiles. Similarly, nanoparticle-adjuvanted TB vaccines, including the M72/AS01E and H56:IC31 candidates, have shown encouraging immunogenicity in human trials. However, several translational bottlenecks persist. The large-scale and reproducible manufacturing of nanocarriers under Good Manufacturing Practice (GMP) conditions remains challenging due to complex physicochemical properties. Regulatory approval pathways for nanomedicines are still evolving, with limited standardized protocols for toxicity, stability, and pharmacokinetic assessments. Additionally, the high production costs, scalability concerns, and cold-chain requirements restrict widespread adoption in low-resource settings where TB burden is highest. Addressing these regulatory, manufacturing, and economic barriers will be critical for accelerating the clinical integration of nanotechnology-based TB therapies.

Furthermore, advancements in nanoparticle-based vaccines and mRNA formulations address the limitations of the BCG vaccine by improving antigen presentation and enhancing protective immune responses. Moving forward, the development of multifunctional nanocarriers capable of co-delivering drugs and imaging agents holds potential for theranostic applications. The expansion of inhalable formulations adapted for children and extrapulmonary TB could enhance treatment accessibility. Clinical translation of CRISPR and host-directed therapies requires rigorous validation but represents a shift towards personalized TB treatment. Scalable production of nanoparticle-based vaccines tailored for high-burden areas could strengthen global immunization efforts. This study underlines that combining nanotherapeutics, gene editing, immune modulation, and targeted delivery represents a comprehensive approach to overcoming resistance, reducing treatment duration, and ultimately accelerating global TB elimination. This revised framework connects specific challenges in TB therapy with corresponding technological innovations, enhancing the logical flow and clarity of discussion. By structuring the review around a problem–solution paradigm, the study provides an integrated understanding of how emerging nanosystems, gene-editing tools, and immunotherapies collectively address the unmet needs in TB management.

## Figures and Tables

**Table 1:** Standard antitubercular therapy (ATT) regimens, active agents, and mechanisms [32].

Category	Drugs/Regimen	Duration & Phase	Mechanism of Action/Target	Remarks
First-Line Drugs (for Drug-Susceptible TB)	Isoniazid (INH), Rifampicin (RIF), Pyrazinamide (PZA), Ethambutol (EMB)	Intensive Phase (2 months): All four drugs Continuation Phase (4 months): INH + RIF	INH—inhibits mycolic acid synthesis; RIF—inhibits RNA polymerase; PZA—disrupts membrane potential; EMB—inhibits arabinogalactan synthesis	Core WHO-recommended regimen; >85% cure rate if adhered properly
Second-Line Drugs (for MDR-TB/XDR-TB)	Fluoroquinolones (Levofloxacin, Moxifloxacin), Aminoglycosides (Amikacin, Kanamycin), Ethionamide, Cycloserine, Linezolid, Clofazimine	Duration varies (18–24 months depending on resistance)	Inhibit DNA gyrase, protein synthesis, or mycolic acid synthesis	Used when resistance to INH/RIF occurs; higher toxicity, cost, and duration
New/Novel Drugs (for MDR/XDR-TB)	Bedaquiline, Delamanid, Pretomanid	6–9 months (as part of BPaL regimen: Bedaquiline + Pretomanid + Linezolid)	Target ATP synthase or inhibit mycolic acid biosynthesis	Effective against highly resistant TB; WHO-endorsed shorter regimen
Adjunct Therapy/Supportive Agents	Pyridoxine (Vitamin B6), Metformin, Statins	Throughout treatment	Neuroprotection (B6), host-directed immunomodulation (Metformin/Statins)	Reduces side effects and enhances the host immune response

**Table 2:** Comparison of Nanocarrier Systems for Tuberculosis Drug Delivery.

Nanocarrier Type	Material	Mechanism/Advantage	Target Site	Drugs Delivered	Reference
Liposomes	Phospholipid bilayer	Mimics the cell membrane, enhances uptake by macrophages	Intracellular macrophages	Rifampicin, Isoniazid	[7,34]
Solid Lipid Nanoparticles (SLNs)	Solid lipids	Improved drug stability, controlled release	Pulmonary site	Rifampicin	[8,35]
Polymeric Nanoparticles	PLGA, Chitosan	Targeted delivery, biodegradable	Granulomas, Macrophages	Multiple anti-TB drugs	[9,36]
Dendrimers	Branched polymers	Multidrug conjugation, high payload	Infected tissues	Drugs + Imaging agents	[10]
Metallic Nanoparticles	Gold, Silver	Intrinsic antimicrobial effects	Bacterial envelope	N/A	[36]

**Table 3:** Emerging Therapies and Mechanisms in TB Treatment.

Therapy Type	Mode of Action	Target	Advantage	Reference
CRISPR-Cas Technology	Gene silencing/editing	MTB virulence/resistance genes	Precision targeting of resistant strains	[14,41]
Host-Directed Therapies (HDTs)	Modulate autophagy, cytokines, and immune signaling	Host immune response	Enhances pathogen clearance, reduces inflammation	[12,13]
Phage Therapy	Lysis of MTB by mycobacteriophages	Drug-resistant *M. tuberculosis*	Effective against MDR/XDR strains	[15]
Nanoparticle-based Vaccines	Improved antigen presentation	Immune system (APCs)	Enhanced cellular immunity, durable protection	[16,43]

**Table 4:** Factors Affecting TB Treatment Outcomes.

Factor	Impact on TB Treatment	Clinical Relevance	Reference
HIV Co-infection	Alters immune response, increases drug interactions	Higher mortality, complex therapy	[89]
Patient Compliance	Irregular dosing leads to resistance	The major cause of MDR-TB	[90]
Pharmacokinetics	Affects drug levels & response	Requires personalized dosing	[91]
Comorbidities (e.g., diabetes, CKD)	Impairs immunity, changes drug metabolism	Requires monitoring & dose adjustments	[92]
Socioeconomic Factors	Poverty and stigma affect adherence & access	Public health barrier	[93]
